# Activity and Interactions of Liposomal Antibiotics in Presence of Polyanions and Sputum of Patients with Cystic Fibrosis

**DOI:** 10.1371/journal.pone.0005724

**Published:** 2009-05-28

**Authors:** Misagh Alipour, Zacharias E. Suntres, Majed Halwani, Ali O. Azghani, Abdelwahab Omri

**Affiliations:** 1 The Novel Drug & Vaccine Delivery Systems Facility, Department of Chemistry and Biochemistry, Laurentian University, Sudbury, Ontario, Canada; 2 Medical Sciences Division, Northern Ontario School of Medicine, Lakehead University, Thunder Bay, Ontario, Canada; 3 Department of Biology, University of Texas at Tyler, Tyler, Texas, United States of America; Abramson Research Center, United States of America

## Abstract

**Background:**

To compare the effectiveness of liposomal tobramycin or polymyxin B against *Pseudomonas aeruginosa* in the Cystic Fibrosis (CF) sputum and its inhibition by common polyanionic components such as DNA, F-actin, lipopolysaccharides (LPS), and lipoteichoic acid (LTA).

**Methodology:**

Liposomal formulations were prepared from a mixture of 1,2-Dimyristoyl-*sn*-Glycero-3-Phosphocholine (DMPC) or 1,2-Dipalmitoyl-*sn*-Glycero-3-Phosphocholine (DPPC) and Cholesterol (Chol), respectively. Stability of the formulations in different biological milieus and antibacterial activities compared to conventional forms in the presence of the aforementioned inhibitory factors or CF sputum were evaluated.

**Results:**

The formulations were stable in all conditions tested with no significant differences compared to the controls. Inhibition of antibiotic formulations by DNA/F-actin and LPS/LTA was concentration dependent. DNA/F-actin (125 to 1000 mg/L) and LPS/LTA (1 to 1000 mg/L) inhibited conventional tobramycin bioactivity, whereas, liposome-entrapped tobramycin was inhibited at higher concentrations - DNA/F-actin (500 to 1000 mg/L) and LPS/LTA (100 to 1000 mg/L). Neither polymyxin B formulation was inactivated by DNA/F-actin, but LPS/LTA (1 to 1000 mg/L) inhibited the drug in conventional form completely and higher concentrations of the inhibitors (100 to 1000 mg/L) was required to inhibit the liposome-entrapped polymyxin B. Co-incubation with inhibitory factors (1000 mg/L) increased conventional (16-fold) and liposomal (4-fold) tobramycin minimum bactericidal concentrations (MBCs), while both polymyxin B formulations were inhibited 64-fold.

**Conclusions:**

Liposome-entrapment reduced antibiotic inhibition up to 100-fold and the CFU of endogenous *P. aeruginosa* in sputum by 4-fold compared to the conventional antibiotic, suggesting their potential applications in CF lung infections.

## Introduction

Chronic bronchial infections caused by opportunistic pathogens in the lower respiratory tract are a major cause of health decline in the CF population [1]. These recurrent infections are mainly due to Gram-negative bacteria, with *P. aeruginosa* being the most common species isolated [2]–[4]. Bacterial infections lead to biofilm formation and host inflammatory responses and the ultimate resistance to antibacterial therapies results in increased morbidity and mortality [5]–[10]. Presently, prophylactic anti-inflammatory and antibacterial chemotherapy have dramatically improved the life span of the CF population, albeit pathogenic resistance to commonly used antibiotics has raised the demand for the development of novel therapeutic modalities [11]–[13].

Antibiotics like aminoglycosides and polymyxins have been used for the treatment of acute or chronic exacerbations in response to multi-drug resistant (MDR) bacteria, particularly Gram-negative bacilli such as *P. aeruginosa* [14]–[17]. Aminoglycosides including tobramycin contain broad antibacterial and post-antibiotic effect, but due to their hydrophilic nature, they are not absorbed and have adverse effects (i.e. nephrotoxicity, ototoxicity) when parenterally administered [17]–[19]. Presently, intravenous administration of aminoglycosides is widely used by CF clinicians and limiting the dose to daily administration seems to reduce adverse effects [20], [21]. Polymyxins are cationic polypeptides that bind to lipopolysaccharide of the Gram-negative bacteria and increase their membrane permeability and cell death. Cytotoxicity issues and adaptive resistance by bacterial cell surface alterations have limited their application to cases where other antibiotics have failed [22]–[24].

Clinical studies have shown the success of antibiotics used in inhalation therapy, alone or in synergism, to combat multi-drug resistant *P. aeruginosa* [25]–[31]. However, loss of innate immune response, the emergence of resistant mucoidal strains, and increase in biofilm production, and the buildup of thick polyanionic sputum have hampered complete eradication of these infections [7], [32]–[35]. Although an antibiotic may display activity against planktonic bacteria *in vitro*, the harsh environment of sputum containing factors produced by host and the microbes reduce their potential interactions with the targeted pathogens [36], [37]. Clinical experiments have shown that in the presence of sputum, antibiotic potency is reduced mainly because of binding to sputum and its inhibitory components like glycoproteins [e.g. mucin (8–47 mg/mL)] [38], neutrophil derived DNA (0.6–6.6 mg/mL) [38], and actin filaments (0.1–5 mg/mL) [39], and bacterial endotoxins such as LPS and LTA [40]–[47].

Liposomes are biodegradable delivery vesicles made up of single or multiple phospholipids in the range of several nanometers to micrometers [48], [49]. It is clear that entrapment of the majority of antibacterial agents in liposomes tends to enhance bioactivity, bioavailability, and lower drug toxicity [50]–[52]. Liposomes may protect the entrapped agent from aggregation and inactivation with polyanionic components of the CF sputum, hence increasing its activity at the site, although the sputum may act as a barrier to larger liposomes [53]–[55]. The present study was carried out to answer the following questions: (i) Are liposome-entrapped antibiotics stable in the environment of the sputum? (ii) Will the entrapment within liposomes reduce antibiotic interaction with the inhibitory factors present in the sputum? (iii) Will liposome-entrapped antibiotics reduce the number of live bacteria in sputum more effectively than the free antibiotics?

Our data demonstrate that liposomes are stable in presence of sputum and inhibitory factors. This data is encouraging as it displays the ability of lipid vesicles to protect the antibiotics from inactivation. The study shows that free tobramycin and polymyxin B, incubated with negatively charged inhibitory factors, is greatly inhibited compared to liposome-entrapped forms at higher concentrations. Liposome-entrapped antibiotics display higher reduction in CFU of endogenous *P. aeruginosa* in sputum compared to the free antibiotic suggesting its potency in CF lung infections.

## Materials and Methods

### F-actin and other chemicals

Human placental DNA, G-actin, *Escherichia coli* (O111:B4) lipopolysaccharide (LPS), and *Staphylococcus aureus* lipoteichoic acid (LTA) were purchased from Sigma Chemicals Co (St. Louis, MO, USA). Monomeric G-actin was prepared from an acetone powder of rabbit skeletal muscle in a non-polymerizing buffer (10 mM TRIS, pH 7.4, 0.2 mM CaCl_2_, 0.2 mM ATP, 1 mM Dithiothreitol). G-actin was then polymerized to F-actin with the addition of 2 mM MgCl_2_ and 150 mM KCl and gently shaken for 1 h at room temperature. Depending on the experiment, DNA, LPS and LTA were dissolved in double distilled H_2_O or in cation-adjusted Mueller-Hinton (CAMH) broth. Synthetic 1,2-Dimyristoyl-*sn*-Glycero-3-Phosphocholine (DMPC), Cholesterol (Chol), and 1,2-Dipalmitoyl-*sn*-Glycero-3-Phosphocholine (DPPC) were obtained from Northern Lipids Inc (Burnaby, BC, Canada). Polymyxin B (Alexis Biochemicals, Burlington, NC, USA), and tobramycin (Sandoz Laboratories, Boucherville, QC, Canada), were diluted in Phosphate Buffered Saline solution (PBS: 160 mM NaCl, 10 mM KH_2_PO_4_, pH 7.4).

### Organisms

Reference strains *P. aeruginosa* (ATCC 27853) were purchased from PML Microbiologicals (Mississauga, ON, Canada). Clinical isolate strains *PA*-48912-1, *PA*-48912-2, and *PA*-48913 were kindly obtained from the Clinical Microbiology Laboratory of Memorial Hospital (Sudbury, ON, Canada) and grown to form biofilm as described elsewhere [56]. The strains were inoculated onto CAMH agar plates and incubated for 18 h at 37°C before any experiments. For any bactericidal experiment involving ATCC 27853, single colonies were suspended to a concentration of 1×10^6^ cfu/mL in CAMH broth before addition to 96-well plates.

### Preparation and characterization of liposomal antibiotics

Liposome- entrapped tobramycin or polymyxin B was prepared from a lipid mixture of either DMPC or DPPC and Chol (molar ratio of 2∶1), respectively, by dehydration-rehydration method as described previously with slight modifications [57], [58]. In brief, lipids were dissolved in chloroform and removed under vacuum at 53°C using a rotary evaporator (Buchi-Rotavapor R205, Brinkmann, Toronto, ON, Canada). 2 ml of an aqueous solution of tobramycin or polymyxin B at a concentration of 10 mg/ml were added to the thin dry lipid film and hand shaken in a warm water bath for 1 minute. The lipid suspensions were sonicated in a round-bottom Erlenmeyer flask for 5 minutes (Sonic Dismembrator Model 500, Fischer Scientific, USA) while submerged in an ice-bath. The sonicator was not in direct contact with the liposome suspension at any time. The suspension was freeze-dried overnight for preservation and higher entrapment (Labconco model 77540, USA). At the time of experiment, dehydrated liposomes were rehydrated in PBS above the phase transition temperature of lipids (DMPC *T_c_* = 23°C; DPPC *T_c_* = 41°C), for 2 h and unentrapped drug was washed off twice by ultracentrifugation at 62000 g. This step ensures that the unentrapped drug (in the supernatant) is separated from the liposomal pellet and is aspirated from the formulation. The liposomal suspensions were diluted at room temperature and size and polydispersity index was automatically determined with the use of a NICOMP 270/autodilute Submicron Particle Sizer according to manufacturer instructions (Santa Barbara, CA, USA). The content of antibiotic entrapped in liposomes (after disruption with 0.2% Triton X-100) was measured by an established method as described previously for tobramycin and polymyxin B [51], [58]. Encapsulation efficiency (EE) was calculated as follows:
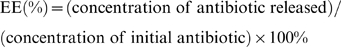



### Stability of liposomes loaded with antibiotics

The stability of antibiotics in the formulations was examined according to Mugabe et al. [59] at 37°C for 18 h in the presence of PBS, CAMH broth, supernatant of biofilm forming *P. aeruginosa* (*PA*-48912-1, *PA*-48912-2, and *PA*-48913), a combination of DNA, F-actin, LPS, and LTA at a concentration of 1000 mg/L, and intact or autoclaved sputum. In experiments involving sputum, pooled CF sputum was either kept intact and diluted 1∶10 (w/v), or autoclaved for 10 min before mixing with CAMH broth. After incubation, aliquots of the mixtures were removed and centrifuged. Antibiotic presence in the pellet was assayed by the microbiological assay as described above, and the amount of antibiotic released from the liposomes was expressed as a percentage of the total antibiotic concentration at 0 h.

### Bacterial killing assays in presence of polyanions

Antibacterial activity of the formulations was measured in the presence or absence of polyanions found in the CF lung. *P. aeruginosa* (ATCC 27853) was grown on CAMH agar overnight at 37°C. Single colonies were diluted and suspended in CAMH broth alone or with 2-fold dilutions of LPS, LTA, DNA and F-actin (125 to 1000 mg/L); 2-fold dilutions of DNA and F-actin (125 to 1000 mg/L); and 10-fold dilutions of LPS and LTA (1 to 1000 mg/L). Equal volumes of 100 µL were added to a 96-well plate to a final concentration of 1×10^6^ cfu/mL. To each well, 100 µL of the free or liposome-entrapped antibiotic (0.125–256 mg/L; final concentration) was added and the plates were incubated for 3 h at 37°C. The incubation period and concentration chosen were adequate to allow liposome or free antibiotic-bacteria interaction and eradication. After incubation, the suspensions were kept cool on ice and bacterial suspensions were diluted 10–10000 folds in PBS. Wells treated in the absence of polyanions were plated as is, i.e., without any dilutions. Aliquots (100 µL) of each dilution were plated on CAMH agar and incubated overnight at 37°C. The cfu/mL values were then determined for each of the three independent experiments.

To determine the ability of liposomes to retain their antibiotic activity, the MBC of the antibiotic formulations were determined in an 18 h period by a standard microbroth dilution assay in CAMH broth alone or with a mixture of LPS, LTA, DNA and F-actin at a fixed concentration of 1000 mg/L. MBC assay was performed as mentioned above with addition of 2-fold dilutions of the free or liposomal antibiotic formulations added to the 96-well plates. The final volume in each well was 200 µL, and PBS or CAMH alone were used as positive (no antibiotic) and negative (no bacteria) controls, respectively. Following incubation for 18 h, aliquots (100 µL) were aspirated from each well and subcultured on CAMH agar plates overnight. MBC was defined as the lowest concentration of the antibiotic that resulted in less than 30 cfu live bacteria/Petri dish.

### Expectorated Sputum

The sputum samples were collected by spontaneous expectoration from nine CF patients following informed consent and a protocol approved by the Research Ethics Committee (Sudbury Regional Hospital, Sudbury, Ontario, Canada). Patients' age, name, treatments and exacerbation records were kept confidential. Sputum samples colonized with moderate to heavy growth of *P. aeruginosa* were pooled and frozen at −80°C in aliquots. To measure the effects of the formulations on endogenous *P. aeruginosa*, aliquots of sputum samples were also stored at 4°C and used within 24 h of collection. At the time of the experiment, the sputum samples were diluted 1∶10 (w/v) in CAMH broth and mixed with the antibiotic formulations to achieve concentrations ranged 1 to 512 mg/L. The mixtures were then incubated for 18 h at 37°C and the cfu/mL of live bacteria was determined according to the aforementioned protocol. The dilution of the samples should have affected the viscoelastic properties of the sputum, and this dilution was only done for easier handling and measurement of the sputum samples.

### Data analysis

All results were expressed as mean±S.E.M. obtained from three trials. Comparisons between free and liposomal formulations were made by ANOVA one-way post *t*-test, and *P*-values were considered significant when (*) *p*<0.05, (**) *p*<0.01, (***) *p*<0.001.

## Results

### Liposome entrapment and sizing

The entrapment efficiency of tobramycin in liposomes composed of DMPC/Chol (35 mg∶ 10 mg) was 2.47±0.19 mg/ml with a mean size of 293.7±41.1 nm, (polydispersity index of 0.70±0.12). Liposomes containing polymyxin B in DPPC/Chol (38 mg∶ 10 mg) had an entrapment efficiency of 0.4±0.02 mg/ml, with a mean size of 445.1±49.3 nm (polydispersity index of 0.91±0.06).

### Stability of liposome-entrapped antibiotics

Liposomal stability and antibiotic leakage in different environments including CF sputum at 3 or 18 h post-exposure are shown in Table 1. The release rate of antibiotics in the presence of bacterial supernatant, polyanionic components, autoclaved or intact sputum was comparable to PBS buffer or CAMH broth controls.

**Table 1:** Liposome-entrapped antibiotic stability assayed by microbiological assay.

Formulations	Conditions	Retention at 3 h	Retention at 18 h
**Liposomal tobramycin**	**PBS buffer**	72.7±3.2%	71.5±2.6%
	**CAMH broth**	74.6±2.1%	73.3±0.5%
	**Bacterial Supernatant**	72.9±1.6%	74.1±2.0%
	**Polyanionic broth**	74.3±2.2%	73.3±1.8%
	**Sterile Sputum**	72.1±3.0%	71.4±1.1%
	**Intact Sputum**	73.8±1.9%	71.7±1.4%
**Liposomal polymyxin B**	**PBS buffer**	67.5±1.3%	54.9±1.8%
	**CAMH broth**	65.2±1.2%	54.7±1.7%
	**Bacterial Supernatant**	67.5±2.1%	54.9±2.7%
	**Polyanionic broth**	69.4±2.7%	51.3±2.4%
	**Sterile Sputum**	67.2±2.6%	53.3±2.4%
	**Intact Sputum**	65.4±1.5%	52.3±1.1%

The stability of the liposomal formulations were examined at 37°C in an 18 h period in the presence of PBS, CAMH broth, supernatant of biofilm forming *P. aeruginosa*, a combination of DNA, F-actin, LPS, and LTA, and diluted intact or autoclaved sputum.

### Effect of DNA, F-actin, LPS, and LTA on bactericidal activity

**Figure 1:**
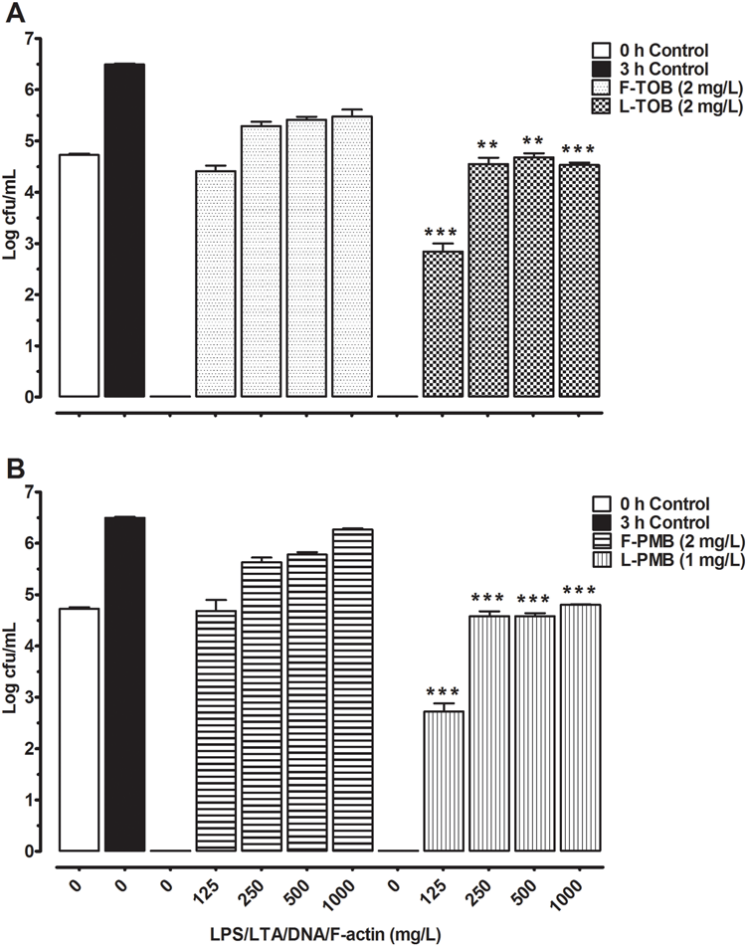
Bactericidal activity and inhibition of antibiotics by DNA, F-actin, LPS and LTA. A) Bactericidal concentrations of free tobramycin (F-TOB) and liposomal tobramycin (L-TOB) were incubated in presence of LPS/LTA (1 to 1000 mg/L). B) Bactericidal concentrations of free polymyxin B (F-PMB) and liposomal polymyxin B (L-PMB) were incubated in presence of DNA/F-actin/LPS/LTA (125 to 1000 mg/L). Growth controls are represented at 0 h (empty bar), and 3 h (dark bar). Comparisons between free and liposomal formulations were made by ANOVA one-way post *t*-test, and *P*-values were considered significant when (**) *p*<0.01, (***) *p*<0.001.

To determine the inhibitory effects of DNA, F-actin, LPS, or LTA on the activity of antibiotics, different concentrations of these inhibitory factors were co-incubated with the antibiotic formulations during a 3 h pre-incubation first. The free and liposomal tobramycin at 2 mg/L killed all ATCC 27853 strain within 3 h, while the liposomal and free polymyxin B eradicated bacteria at 1 mg/L and 2 mg/L, respectively. The bactericidal activity for both groups of antibiotics in the presence of DNA, F-actin, LPS, and LTA at the concentrations of 125 to 1000 mg/L is shown in Figure 1. The activities of free and liposomal antibiotics were strongly inhibited and liposomal formulations tended to display lower antibiotic inhibition.

**Figure 2:**
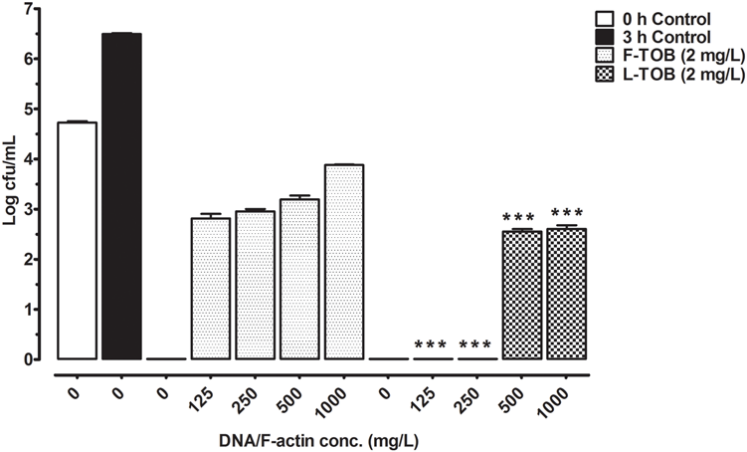
Bactericidal activity and inhibition of tobramycin by DNA and F-actin. Bactericidal concentrations of free tobramycin (F-TOB), and liposomal tobramycin (L-TOB) at 2 mg/L were incubated with *P. aeruginosa* (ATCC 27853), or in presence of DNA/F-actin (125 to 1000 mg/L). Growth controls are represented at 0 h (empty bar), and 3 h (dark bar). Comparisons between free and liposomal tobramycin was made by ANOVA one-way post *t*-test, and *P*-values were considered significant when (***) *p*<0.001.

**Figure 3:**
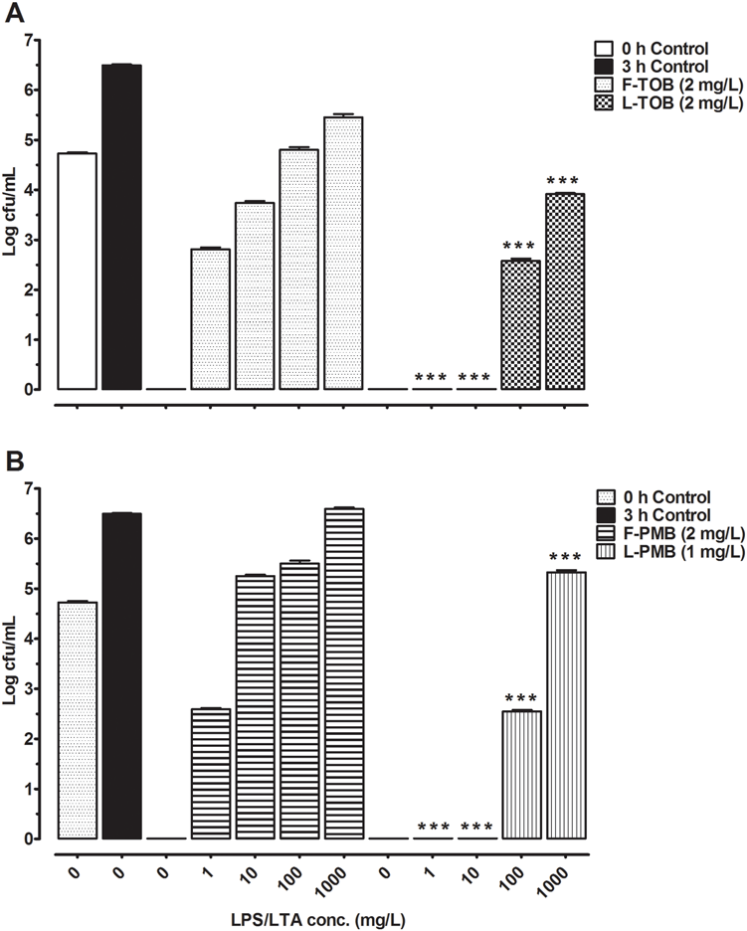
Bactericidal activity and inhibition of antibiotics by LPS and LTA. A) Bactericidal concentrations of free tobramycin (F-TOB) and liposomal tobramycin (L-TOB) were incubated in presence of LPS/LTA (1 to 1000 mg/L). B) Bactericidal concentrations of free polymyxin B (F-PMB) and liposomal polymyxin B (L-PMB) were incubated in presence of LPS/LTA (1 to 1000 mg/L). Growth controls are represented at 0 h (empty bar), and 3 h (dark bar). Comparisons between free and liposomal formulations were made by ANOVA one-way post *t*-test, and *P*-values were considered significant when (***) *p*<0.001.

Separately, the inhibition of activity by DNA and F-actin, LPS and LTA were assessed. When DNA and F-actin were co-incubated with the tobramycin formulations (Figure 2), free tobramycin failed to eradicate growth at DNA/F-actin concentrations of 125 to 1000 mg/L. Higher concentrations of these inhibitory factors (500 to 1000 mg/L) however, were required to hinder the liposomal tobramycin activity. On the other hand, bactericidal activity of liposomal polymyxin B co-incubated with DNA/F-actin remained the same as antibacterial activity was not impaired within 3 h (data not shown). Under the same conditions discussed above, the effects of bacterial surface components LPS and LTA on the activity of antibiotics were investigated. Free tobramycin (Figure 3A) activity was increasingly inhibited at LPS/LTA concentrations of 1 to 1000 mg/L. While the lower concentrations (1 to 10 mg/L) did not have any effect, higher concentrations (100 to 1000 mg/L) of LPS/LTA were able to inactivate liposomal tobramycin. Polymyxin B formulations behaved the same as tobramycin in the presence of LPS/LTA, as indicated in Figure 3B.

Since negatively charged polyanions hindered bactericidal activity in a short period of time (3 h exposure), their effect on the MBCs in an 18 h period were also investigated (Table 2). MBC levels increased 16-fold for free tobramycin (16 mg/L) compared to 4-fold for its liposomal form (8 mg/L). Free (32 mg/L) and liposomal polymyxin B (16 mg/L) were inhibited equally by the polyanions (64-fold increase in MBC).

**Table 2:** Minimum Bactericidal Concentrations.

Formulations	MBC (mg/L)	
	CAMH broth	Inhibitory factors
Free tobramycin	1	16
Lipo tobramycin	2	8
Free polymyxin B	0.5	32
Lipo polymyxin B	0.25	16

Bactericidal activity of free and liposomal formulations against susceptible *P. aeruginosa* ATCC 27853 strain was carried out in broth alone or in presence of DNA/F-actin/LPS/LTA at a final concentration of 1000 mg/L.

### Antibacterial activity on CF sputum

**Figure 4:**
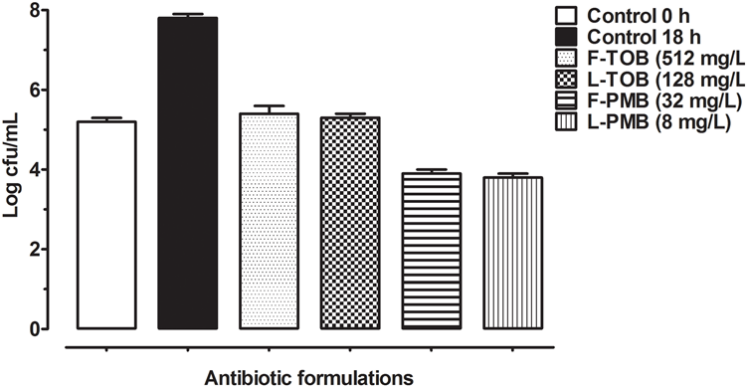
CF Sputum treatment with various antibiotic formulations. CFU counts were made after incubation of diluted CF sputum (1∶10 w/v) in PBS with two-fold dilutions of free tobramycin at 512 mg/L (F-TOB), liposomal tobramycin at 128 mg/L (L-TOB), free polymyxin B at 32 mg/L (F-PMB), and liposomal polymyxin B at 8 mg/L (L-PMB). Growth controls are represented at 0 h (empty bar), and 18 h (dark bar).

To test the efficacy of entrapped versus free antibiotics in the CF sputa, pooled sputum was diluted and incubated with increasing concentrations of tobramycin and polymyxin B for 18 h. As shown in Figure 4, bacterial counts were reduced, but neither of the formulations eradicated endogenous bacteria present in the sputum. Liposomal tobramycin (128 mg/L; 5.3±0.1 logs) and polymyxin B (8 mg/L; 3.8±0.1 logs) displayed higher bactericidal activity than free tobramycin (512 mg/L; 5.4±0.2 logs) and polymyxin B (32 mg/L; 3.9±0.1 logs). The sputum itself did not seem to have any antibacterial activity against endogenous strains as bacterial counts were increased from 0 h (5.2±0.1 logs) to 18 h (7.8±0.1 logs).

## Discussion

Polycationic antibacterial agents, like aminoglycosides and polymyxins, require self-promoted uptake pathways for entry and eradication of Gram-negative bacteria [60]. The cationic antibiotics increase bacterial outer membrane permeability by displacing magnesium ions and binding to LPS [41], [61]. In the highly ionic CF sputum, however, the high affinity of excreted polyanionic bacterial endotoxins and glycoproteins from lysed white blood cells towards cationic antibiotics decreases their overall interaction with the bacteria in the lungs [46], [62]. Liposomes may create a protective environment for antibacterial agents to minimize such interactions and subsequently maintain a steady drug concentration in the lungs. Our data on the stability of the liposomal formulations displays that tobramycin leakage was at equilibrium after 3 h, while polymyxin B leakage continued up to half its concentration over 18 h. This suggests that these nanoparticles are effective in protecting the antibiotics in the CF sputum *in vitro*. The stability will ensure a continuous presence of the antibiotic at the site of infection, and improves antibiotic bioavailability and biodistribution *in vivo* [63].

Polyanions like DNA and F-actin have strong affinity for their multivalent counterions and tend to aggregate (form bundles) in the presence of cationic antibiotics which block their bioactivity [40], [46], [64], [65]. Our results demonstrate the capability of liposomes to reduce the antibiotics' contact with polyanionic factors in the sputum and enhance bacteria-antibiotic(s) interactions. The liposomal formulation protected tobramycin from the inhibitory actions of DNA/F-actin at low concentrations while neither polymyxin B formulations were inactivated. Our findings are in agreement with those reported by Hunt *et al.* [36] who found a reduction in tobramycin activity in the presence of DNA (within a 2 h exposure) even when it was pretreated with recombinant human DNase (rhDNase). Weiner *et al.* [43] on the other hand, reported DNA and F-actin aggregation (within a 5 h exposure) with increasing concentrations of tobramycin, yet bioactivity in a microbroth dilution assay (within an 18 h exposure) was not hindered by the presence of either DNA or F-actin. The inconsistencies among the results of the different studies may be attributed to factors such as incubation time, co-incubation of DNA and F-actin, and that DNA/F-actin concentrations were increased as tobramycin concentration was kept constant. The protective effect of the liposomes at the lower DNA/F-actin concentrations may be attributed to the neutral nature of the phospholipids comprising the liposomes which would not favor electrostatic interactions between phospholipids with DNA or F-actin. The lack of effectiveness of tobramycin encapsulated within liposomes in the presence of the higher concentrations of polyanionic factors cannot be explained from the results of this study but it may be possible that a build up of F-actin/DNA aggregates leads to an increase in viscoelasticity, which ultimately hinders liposome-bacteria interaction [64], [65]. Reports from other studies have shown that DNA greatly hampers nanosphere diffusion through sputum and that the rhDNase improves its diffusion [53], [55], [66].

With regards to polymyxin B, reports from studies have shown G-actin polymerization in the presence of polymyxin B [37] and DNA and polymyxin B precipitation *in vitro* [67]. In our studies, there was no loss of bioactivity when DNA, F-actin, or both were incubated with polymyxin B (data not shown). The observation of consistent bacterial killing by polymyxin B can be attributed to the ability of the antibiotic to resist bundle formation, and having a higher affinity for polyanionic LPS of the bacterial outer wall than DNA or F-actin. Weiner *et al.* [43] reported no aggregation or reduction of bioactivity between colymycin, an anionic colistin form, and DNA or F-actin. However, the absence of aggregation may be due to the similar negative charges of the antibiotic and DNA or F-actin.

The binding of free bacterial surface components (e.g. LPS and LTA) to polycationic antibiotics like polymyxin B may be beneficial to the host in terms of suppressing inflammation however it will compromise the antibacterial effect of the antibiotic. Tobramycin and polymyxin B tend to interact with the bacterial lipid membranes as indicated by the results of this study where the bioactivity of both antibiotics was reduced when co-incubated with LPS/LTA. However, the bioactivity of the antibiotics within the liposomes fared better (Figure 3) although inhibited at the higher LPS/LTA concentrations. The mechanism of inactivation of liposomal antibiotics by the higher polyanionic LPS/LTA levels cannot be attributed to the release of antibiotics from liposomes and subsequent inactivation because results from the liposomal stability studies (Table 1) showed that the lipid bilayers were not lysed. This is consistent with results from another study reported by Davies *et al.* [68] where divalent anions entrapped in negative or positive charged liposomes when incubated with LPS were not significantly leaked from the liposomes which were not lysed. It is possible then that the higher concentrations of LPS/LTA may contribute to the stabilization of the liposomes, reduce antibiotic release, and thus prevent the leakage of the antibiotics leading to reduction of their interaction with bacteria.

If the lipid bilayers of liposomes can decrease antibiotic interactions with the polyanionic components found in CF lungs and reduce bacterial growth within a 3 h period much more strongly than free antibiotics, its long term advantage and presence in an 18 h period would be advantageous (Table 2). Unfortunately, prolonged contact between polyanions and the formulations greatly increased the free and liposomal polymyxin B bactericidal concentrations, with liposomal tobramycin exhibiting better activity than free tobramycin. The dissimilar inhibitory effects on tobramycin and polymyxin B may be attributed to differences in their mechanisms of action, as tobramycin, a polar drug can enter the cell while polymyxin B a lipophilic agent interacts with LPS on the cellular surface. The interaction of polymyxin B with cell surface LPS, in addition to the interaction with the polyanions might leads to competition at the LPS binding site of bacteria, ultimately reducing antibiotic binding.

In light of the higher bactericidal activities and lower inactivation of liposomal antibiotics in the presence of polyanionic components *in vitro*, we sought to compare the bactericidal activity of these formulations against endogenous *P. aeruginosa* in CF sputa to that of the free drug. As shown in Figure 4, the antibacterial activity of liposomal antibiotics was more effective than the free antibiotics by 4-fold, although due to a large microbial population in the CF sputum, neither of the formulations fully eradicated bacterial growth. While liposomal tobramycin (128 mg/L) reduced growth, liposomal polymyxin B (8 mg/L) fell into clinically acceptable levels. The high concentrations of antibiotics, tobramycin in particular, required to lower growth, may be primarily due to samples containing antibiotic resistant strains, or the sputum and its contents impeding antibiotic effects by acting as a physical barrier or inhibitor. Several studies have dealt with the inhibitory properties of sputum on antibiotics [34], [42], [69] while there have been a limited number of studies focused on liposomal penetration and interaction with sputum [53]–[55], [70]. The majority of these studies have focused on gene therapy and their transport across the sputum, but a recent work by Meers *et al.* [54] showed the ability of labeled neutral liposomes to penetrate sputum, and furthermore, aminoglycosidic amikacin-entrapped liposomes were more efficacious than free amikacin in reducing bacterial growth in a rat *P. aeruginosa* infection model. In our study, due to issues of confidentiality, we did not have access as to the clinical status of the patients or their pathology laboratory reports. Nevertheless, delivery of antibiotics via a liposomal system enhanced their antibacterial activity in sputum.

Although liposome entrapment of antibiotics and their increased efficacy is not a novel finding, neutral liposome-entrapped antibiotics tended to be more bactericidal in sputum and in the presence of sputum components when compared to free antibiotics, but with reduced efficacies over a longer period of time *in vitro* (18 h exposure). This decrease in efficacy appears to be the result of pro-longed interactions of the liposomes with the polyanionic factors found in sputum. As prophylactic and anti-inflammatory treatments are improving the lung function of CF patients, reduction in neutrophil inflammatory response and bacterial infections may reduce its lysis and the presence of charged macromolecules which tend to inactivate cationic antibiotics. As novel approaches proceed towards a cure for CF, research must also be directed on strategies that obstruct the presence and/or action of inhibitory factors associated with the disease. Future work in our laboratory will tend to focus on disruption of these negatively charged factors for increased liposomal penetration.

## References

[1] Boyle MP (2007). Adult cystic fibrosis.. JAMA.

[2] Son MS, Matthews WJ, Kang Y, Nguyen DT, Hoang TT (2007). In vivo evidence of *Pseudomonas aeruginosa* nutrient acquisition and pathogenesis in the lungs of cystic fibrosis patients.. Infect Immun.

[3] Remmington T, Jahnke N, Harkensee C (2007). Oral anti-pseudomonal antibiotics for cystic fibrosis.. Cochrane Database Syst Rev.

[4] Lahiri T (2007). Approaches to the treatment of initial *Pseudomonas aeruginosa* infection in children who have cystic fibrosis.. Clin Chest Med.

[5] Courtney JM, Ennis M, Elborn JS (2004). Cytokines and inflammatory mediators in cystic fibrosis.. J Cyst Fibros.

[6] Kirov SM, Webb JS, O'May CY, Reid DW, Woo JK (2007). Biofilm differentiation and dispersal in mucoid *Pseudomonas aeruginosa* isolates from patients with cystic fibrosis.. Microbiology.

[7] Murray TS, Egan M, Kazmierczak BI (2007). *Pseudomonas aeruginosa* chronic colonization in cystic fibrosis patients.. Curr Opin Pediatr.

[8] Sagel SD, Chmiel JF, Konstan MW (2007). Sputum biomarkers of inflammation in cystic fibrosis lung disease.. Proc Am Thorac Soc.

[9] Elizur A, Cannon CL, Ferkol TW (2008). Airway inflammation in cystic fibrosis.. Chest.

[10] Driscoll JA, Brody SL, Kollef MH (2007). The epidemiology, pathogenesis and treatment of *Pseudomonas aeruginosa* infections.. Drugs.

[11] Iredell JR (2007). Optimizing antipseudomonal therapy in critical care.. Semin Respir Crit Care Med.

[12] Merlo CA, Boyle MP, Diener-West M, Marshall BC, Goss CH (2007). Incidence and risk factors for multiple antibiotic-resistant *Pseudomonas aeruginosa* in cystic fibrosis.. Chest.

[13] Davies JC, Rubin BK (2007). Emerging and unusual gram-negative infections in cystic fibrosis.. Semin Respir Crit Care Med.

[14] Eisenberg J, Pepe M, Williams-Warren J, Vasiliev M, Montgomery AB (1997). A comparison of peak sputum tobramycin concentration in patients with cystic fibrosis using jet and ultrasonic nebulizer systems. Aerosolized Tobramycin Study Group.. Chest.

[15] Hermann T (2007). Aminoglycoside antibiotics: old drugs and new therapeutic approaches.. Cell Mol Life Sci.

[16] Westerman EM, De Boer AH, Le Brun PP, Touw DJ, Roldaan AC (2007). Dry powder inhalation of colistin in cystic fibrosis patients: a single dose pilot study.. J Cyst Fibros.

[17] Kaye D (2004). Current use for old antibacterial agents: polymyxins, rifampin, and aminoglycosides.. Infect Dis Clin North Am.

[18] Cannella CA, Wilkinson ST (2006). Acute renal failure associated with inhaled tobramycin.. Am J Health Syst Pharm.

[19] Swan SK (1997). Aminoglycoside nephrotoxicity.. Semin Nephrol.

[20] Fernandes B, Plummer A, Wildman M (2008). Duration of intravenous antibiotic therapy in people with cystic fibrosis.. Cochrane Database Syst Rev.

[21] Touw DJ, Knox AJ, Smyth A (2007). Population pharmacokinetics of tobramycin administered thrice daily and once daily in children and adults with cystic fibrosis.. J Cyst Fibros.

[22] Falagas ME, Kasiakou SK (2005). Colistin: the revival of polymyxins for the management of multidrug-resistant gram-negative bacterial infections.. Clin Infect Dis.

[23] Zavascki AP, Goldani LZ, Li J, Nation RL (2007). Polymyxin B for the treatment of multidrug-resistant pathogens: a critical review.. J Antimicrob Chemother.

[24] Macfarlane EL, Kwasnicka A, Ochs MM, Hancock RE (1999). PhoP-PhoQ homologues in *Pseudomonas aeruginosa* regulate expression of the outer-membrane protein OprH and polymyxin B resistance.. Mol Microbiol.

[25] Sobieszczyk ME, Furuya EY, Hay CM, Pancholi P, Della-Latta P (2004). Combination therapy with polymyxin B for the treatment of multidrug-resistant Gram-negative respiratory tract infections.. J Antimicrob Chemother.

[26] Chuchalin A, Csiszer E, Gyurkovics K, Bartnicka MT, Sands D (2007). A formulation of aerosolized tobramycin (Bramitob) in the treatment of patients with cystic fibrosis and *Pseudomonas aeruginosa* infection: a double-blind, placebo-controlled, multicenter study.. Paediatr Drugs.

[27] Govan J (2002). TOBI: reducing the impact of pseudomonal infection.. Hosp Med.

[28] Geller DE, Konstan MW, Smith J, Noonberg SB, Conrad C (2007). Novel tobramycin inhalation powder in cystic fibrosis subjects: pharmacokinetics and safety.. Pediatr Pulmonol.

[29] Lenoir G, Antypkin YG, Miano A, Moretti P, Zanda M (2007). Efficacy, safety, and local pharmacokinetics of highly concentrated nebulized tobramycin in patients with cystic fibrosis colonized with *Pseudomonas aeruginosa*.. Paediatr Drugs.

[30] Horianopoulou M, Lambropoulos S, Papafragas E, Falagas ME (2005). Effect of aerosolized colistin on multidrug-resistant *Pseudomonas aeruginosa* in bronchial secretions of patients without cystic fibrosis.. J Chemother.

[31] Hodson ME, Gallagher CG, Govan JR (2002). A randomised clinical trial of nebulised tobramycin or colistin in cystic fibrosis.. Eur Respir J.

[32] Rogan MP, Taggart CC, Greene CM, Murphy PG, O'Neill SJ (2004). Loss of microbicidal activity and increased formation of biofilm due to decreased lactoferrin activity in patients with cystic fibrosis.. J Infect Dis.

[33] Walker TS, Tomlin KL, Worthen GS, Poch KR, Lieber JG (2005). Enhanced *Pseudomonas aeruginosa* biofilm development mediated by human neutrophils.. Infect Immun.

[34] Ramphal R, Lhermitte M, Filliat M, Roussel P (1988). The binding of anti-pseudomonal antibiotics to macromolecules from cystic fibrosis sputum.. J Antimicrob Chemother.

[35] Davis SD, Bruns WT (1978). Effects of sputum from patients with cystic fibrosis on the activity in vitro of 5 antimicrobial drugs on *Pseudomonas aeruginosa*.. Am Rev Respir Dis.

[36] Hunt BE, Weber A, Berger A, Ramsey B, Smith AL (1995). Macromolecular mechanisms of sputum inhibition of tobramycin activity.. Antimicrob Agents Chemother.

[37] Someya A, Tanaka N (1979). Interaction of aminoglycosides and other antibiotics with actin.. J Antibiot (Tokyo).

[38] Sanders NN, Van Rompaey E, De Smedt SC, Demeester J (2001). Structural alterations of gene complexes by cystic fibrosis sputum.. Am J Respir Crit Care Med.

[39] Vasconcellos CA, Allen PG, Wohl ME, Drazen JM, Janmey PA (1994). Reduction in viscosity of cystic fibrosis sputum in vitro by gelsolin.. Science.

[40] Kater A, Henke MO, Rubin BK (2007). The role of DNA and actin polymers on the polymer structure and rheology of cystic fibrosis sputum and depolymerization by gelsolin or thymosin beta 4.. Ann N Y Acad Sci.

[41] Marshall AJ, Piddock LJ (1994). Interaction of divalent cations, quinolones and bacteria.. J Antimicrob Chemother.

[42] Levy J, Smith AL, Kenny MA, Ramsey B, Schoenknecht FD (1983). Bioactivity of gentamicin in purulent sputum from patients with cystic fibrosis or bronchiectasis: comparison with activity in serum.. J Infect Dis.

[43] Weiner DJ, Bucki R, Janmey PA (2003). The antimicrobial activity of the cathelicidin LL37 is inhibited by F-actin bundles and restored by gelsolin.. Am J Respir Cell Mol Biol.

[44] Landry RM, An D, Hupp JT, Singh PK, Parsek MR (2006). Mucin-*Pseudomonas aeruginosa* interactions promote biofilm formation and antibiotic resistance.. Mol Microbiol.

[45] Fabretti F, Theilacker C, Baldassarri L, Kaczynski Z, Kropec A (2006). Alanine esters of enterococcal lipoteichoic acid play a role in biofilm formation and resistance to antimicrobial peptides.. Infect Immun.

[46] Bucki R, Sostarecz AG, Byfield FJ, Savage PB, Janmey PA (2007). Resistance of the antibacterial agent ceragenin CSA-13 to inactivation by DNA or F-actin and its activity in cystic fibrosis sputum.. J Antimicrob Chemother.

[47] Lethem MI, James SL, Marriott C, Burke JF (1990). The origin of DNA associated with mucus glycoproteins in cystic fibrosis sputum.. Eur Respir J.

[48] Mozafari MR, Flanagan J, Matia-Merino L, Awati A, Omri A, Suntres ZE (2006). Recent trends in the lipid-based nanoencapsulation of antioxidants and their role in foods.. J Sci Food Agric.

[49] Mozafari MR, Johnson C, Hatziantoniou S, Demetzos C (2008). Nanoliposomes and Their Applications in Food Nanotechnology.. J Liposome Res.

[50] Halwani M, Mugabe C, Azghani AO, Lafrenie RM, Kumar A (2007). Bactericidal efficacy of liposomal aminoglycosides against *Burkholderia cenocepacia*.. J Antimicrob Chemother.

[51] Omri A, Suntres ZE, Shek PN (2002). Enhanced activity of liposomal polymyxin B against *Pseudomonas aeruginosa* in a rat model of lung infection.. Biochem Pharmacol.

[52] Allison SD (2007). Liposomal drug delivery.. J Infus Nurs.

[53] Sanders NN, Van Rompaey E, De Smedt SC, Demeester J (2002). On the transport of lipoplexes through cystic fibrosis sputum.. Pharm Res.

[54] Meers P, Neville M, Malinin V, Scotto AW, Sardaryan G (2008). Biofilm penetration, triggered release and in vivo activity of inhaled liposomal amikacin in chronic *Pseudomonas aeruginosa* lung infections.. J Antimicrob Chemother.

[55] Sanders NN, De Smedt SC, Van Rompaey E, Simoens P, De Baets F (2000). Cystic fibrosis sputum: a barrier to the transport of nanospheres.. Am J Respir Crit Care Med.

[56] Ceri H, Olson ME, Stremick C, Read RR, Morck D (1999). The Calgary Biofilm Device: new technology for rapid determination of antibiotic susceptibilities of bacterial biofilms.. J Clin Microbiol.

[57] Alipour M, Halwani M, Omri A, Suntres ZE (2008). Antimicrobial effectiveness of liposomal polymyxin B against resistant Gram-negative bacterial strains.. Int J Pharm.

[58] Mugabe C, Halwani M, Azghani AO, Lafrenie RM, Omri A (2006). Mechanism of enhanced activity of liposome-entrapped aminoglycosides against resistant strains of *Pseudomonas aeruginosa*.. Antimicrob Agents Chemother.

[59] Mugabe C, Azghani AO, Omri A (2006). Preparation and characterization of dehydration-rehydration vesicles loaded with aminoglycoside and macrolide antibiotics.. Int J Pharm.

[60] Nicas TI, Hancock RE (1980). Outer membrane protein H1 of *Pseudomonas aeruginosa*: involvement in adaptive and mutational resistance to ethylenediaminetetraacetate, polymyxin B, and gentamicin.. J Bacteriol.

[61] Hancock RE (2001). Cationic peptides: effectors in innate immunity and novel antimicrobials.. Lancet Infect Dis.

[62] Kharitonov SA, Sjobring U (2007). Lipopolysaccharide challenge of humans as a model for chronic obstructive lung disease exacerbations.. Contrib Microbiol.

[63] Schiffelers R, Storm G, Bakker-Woudenberg I (2001). Liposome-encapsulated aminoglycosides in pre-clinical and clinical studies.. J Antimicrob Chemother.

[64] Bucki R, Byfield FJ, Janmey PA (2007). Release of the antimicrobial peptide LL-37 from DNA/F-actin bundles in cystic fibrosis sputum.. Eur Respir J.

[65] Tang JX, Wen Q, Bennett A, Kim B, Sheils CA (2005). Anionic poly(amino acid)s dissolve F-actin and DNA bundles, enhance DNase activity, and reduce the viscosity of cystic fibrosis sputum.. Am J Physiol Lung Cell Mol Physiol.

[66] Broughton-Head VJ, Smith JR, Shur J, Shute JK (2007). Actin limits enhancement of nanoparticle diffusion through cystic fibrosis sputum by mucolytics.. Pulm Pharmacol Ther.

[67] Potter JL, Matthews LW, Spector S, Lemm J (1965). Complex formation between basic antibiotics and deoxyribonucleic acid in human pulmonary secretions.. Pediatrics.

[68] Davies M, Stewart-Tull DE, Jackson DM (1978). The binding of lipopolysaccharide from Escherichia coli to mammalian cell membranes and its effect on liposomes.. Biochim Biophys Acta.

[69] Bataillon V, Lhermitte M, Lafitte JJ, Pommery J, Roussel P (1992). The binding of amikacin to macromolecules from the sputum of patients suffering from respiratory diseases.. J Antimicrob Chemother.

[70] Stern M, Caplen NJ, Browning JE, Griesenbach U, Sorgi F (1998). The effect of mucolytic agents on gene transfer across a CF sputum barrier in vitro.. Gene Ther.

